# Exploring the hidden mental health consequences of malaria beyond the fever

**DOI:** 10.3389/fnhum.2024.1432441

**Published:** 2024-07-18

**Authors:** Prerana Nandish, Shrinivasa B. M., Sujith Nath N., G. Shankar, Praveen Kumar Tripathi, Himani Kashyap, Animesh Jain, Anup Anvikar, Vani H. Chalageri

**Affiliations:** ^1^Indian Council of Medical Research, National Institute of Malaria Research, Bengaluru, Karnataka, India; ^2^Indian Council of Medical Research, National Institute of Malaria Research, Ranchi, Jharkhand, India; ^3^Department of Clinical Psychology, National Institute of Mental Health and Neurosciences, Bangalore, India; ^4^Department of Community Medicine, Kasturba Medical College Mangalore, Manipal Academy of Higher Education, Karnataka, Manipal, India; ^5^Indian Council of Medical Research, National Institute of Malaria Research, Dwarka, New Delhi, India; ^6^Associate Professor, The Academy of Scientific and Innovative Research, AcSIR, India

**Keywords:** malaria, malaria morbidity, mental health, cerebral malaria, cognitive impairment, neurological impairment, neurocognition

## Abstract

Malaria morbidity has various presentations and the focus now shifts to uncommon signs and symptoms of malaria infection such as cognitive impairment to address the morbidity when the mortality declines. About 50% of children admitted to hospitals due to malaria experience neurological complications due to factors like low blood sugar, inflammation, elevated pressure, decreased oxygen levels, and excitotoxicity. Malaria during pregnancy negatively also impacts children’s cognitive, behavioral, and executive function leading to neurodevelopmental delay due to increased susceptibility which can significantly affect maternal and child health, leading to higher rates of underestimated factors like anxiety, depression, and PTSD. Despite having the world’s second-largest tribal population, India’s indigenous and tribal communities and their mental health are less explored and less understood. Western psychological tools and neurocognitive assessment tools are not universally applicable, thus necessitating the development of tailored tools to investigate psychological or neurocognitive impairment. This paper has illuminated the hidden mental health consequences of malaria infection, emphasizing the prevalence, nature, and implications of psychological distress among affected individuals. The findings underscore the importance of recognizing and addressing these psychological consequences in the holistic management and prevention of malaria and its mental health consequences.

## Methods

### Search strategy

To identify relevant studies, a systematic search was conducted across Google Scholar, Springer, Elsevier and PubMed. The following search terms and keywords were used: “Malaria,” “cerebral malaria,” “psychological impact,” “mental health,” “cognitive functions,” and “neurological impairment.” The search was limited to articles published in English between 2019 and 2023. A total of 90 papers were reviewed.

### Selection criteria

Studies included in this review met the following criteria:

- Examined Malaria patients, survivors, or communities affected by malaria.
- Presented primary data on psychological outcomes post-malaria infection.
- Focused on the psychological and neurological impact of malaria infection.

## Introduction

Approximately 6,08,000 malaria deaths were recorded worldwide in 2022, with India and Indonesia accounting for about 94% of all malaria deaths in the South East Asia region (World Health Organization, 2024). As the disease heads toward elimination (Singh et al., 2024), the focus now shifts to uncommon and lesser known signs and symptoms of malaria infection such as cognitive impairment (GBD 2021 Nervous System Disorders Collaborators, 2024) to address the morbidity when the mortality declines. In the past 15 years, malaria’s global burden has risen from infection and mortality to neurologic and cognitive impairment, necessitating further research, prevention, and treatment, especially in regions where neurodiagnostic resources are scarce and the disease is endemic, where confirming the diagnosis of cerebral malaria (CM) is a challenging task (Mung’Ala-Odera et al., 2004; Birbeck et al., 2010; Ranjha and Sharma, 2021). Although in CM, the severity of neurological impairment may stem from suppressed gene activity leading to cellular dysfunction, axonal issues, disrupted signaling pathways, and ultimately neurodegeneration due to inflammation and cellular stress; it is not limited to severe cases, affecting even non-cerebral severe malaria and mild malaria (Karikari et al., 2021). Post-CM, developmental, cognitive, and behavioral deficits in sub-Saharan Africa can persist for up to a year contributed by factors like HIV co-infection, stature, and MRI abnormalities which are a significant burden on families and communities due to limited resources for treatment, education, and rehabilitation (Langfitt et al., 2019). Children under the age of five, who account for 67% of all fatalities worldwide, bear the greatest burden of severe malaria (Varo et al., 2020) including the rising resistance to insecticides and antimalarials (Wicht et al., 2020; Shahbodaghi and Rathjen, 2022; Suh et al., 2023) and mental health risks leading to neurological impairment (Goyal et al., 2023), necessitate a comprehensive malaria elimination program involving vector control (Ogunah et al., 2020), chemoprevention, vaccination, improved and early diagnosis accuracy (Pradhan et al., 2019), neuroprotective agents (Arthur et al., 2022), neurorehabilitation (Boubour et al., 2020) and regionally tailored strategies to address mental health consequences.

## Burden of malaria

In 2022, malaria cases worldwide increased by 5 million compared to the previous year, with India responsible for 66% of these cases, and 233 million malaria cases were reported in the WHO African Region, constituting 94% of the global total (World Health Organization, 2024). However, there was a 25% increase in malaria cases in the WHO South-East Asia Region between 2021 and 2022, and 35.4 million pregnancies were reported in the WHO African Region, with 36% of them associated with malaria during pregnancy. The WHO observed a 72.3% reduction in indigenous malaria cases from 2010 to 2022. *Plasmodium falciparum* is the primary cause of malaria, causing over 99% of cases in Africa (Varo et al., 2020) with 25 million pregnant women facing *P. falciparum* infection annually, with one in four showing signs of placental infection which despite efforts to improve healthcare quality, can lead to death or disability from preventable or treatable illnesses, deformities, and injuries (Goyal et al., 2023). Nonetheless, funding for malaria research and development (R&D) declined for the fourth consecutive year, particularly in vaccines and basic research, while medicines continued to receive the largest share of funding (Desai et al., 2007; World Health Organization, 2024).

## Mechanism of psychological and neurological complications of malaria

Malaria progresses to CM due to the interaction between host and parasite proteins, especially during blood-stage infection where the parasite reproduces and matures, due to the activation of endothelial cells by parasite-derived molecules (Lima et al., 2021) causing its proteins to appear on infected red blood cells (iRBCs), which allows them to interact with brain endothelium cells. This promotes the attachment of iRBCs to small blood vessels, leading to the breakdown of the BBB (blood brain barrier) and brain inflammation (Tunon-Ortiz and Lamb, 2019) leading to severe pathologies such as inflammation, capillary congestion, and dysregulated coagulation. This among individuals exposed to *P. falciparum,* with tissue ischemia, could lead to neurological impairments, including hearing, cognition, language, visual and motor coordination (Cartwright, 1972; Beare, 2024). The neuroinflammation in the brain triggered by malaria also leads to neural damage and cognitive impairments due to hemozoin production during infection (Velagapudi et al., 2019). Understanding this process is crucial for brain-related issues as psychological distress caused by malaria infection among affected individuals due to immune and nervous system alterations influenced by stimuli and pro-inflammatory or anti-inflammatory responses in malaria cases can be exacerbated by fear of relapse, severe illness, trauma, and economic strain with untreated malaria persisting for 3–5 years, with recurring episodes determined by underlying infection factors (Ouma et al., 2021; White, 2022; Malaria, 2023).

The interconnected immune and nervous systems and exogenous stimuli like infectious agents and vaccines can alter the immune system’s structure and function which can impact the functioning of the central nervous system (CNS) and cognitive behavioral responses (de Sousa et al., 2023). MRI and fluorescence angiography have been linked to severe cases of malarial retinopathy, longer unconsciousness periods, and neurological issues (MacCormick et al., 2022). A 2010 Ugandan study found that children with childhood malaria had retinopathy, severe hearing impairment, aphasia, dysarthria, motor deficits, movement disorders, gait disorders, behavior problems, and bilateral temporal lobe atrophy (Idro et al., 2010). Children with malaria retinopathy show significant clinical and laboratory differences compared to those without retinopathy, meeting the standard clinical case definition for CM. Early detection, intervention, and long-term follow-up studies are crucial for improving pediatric CM outcomes. Including malaria retinopathy in diagnostic criteria may enhance accuracy and prevent pre-existing CNS injuries as it is an *in vivo* perspective on the brain pathophysiology of CM that is not achievable by any other means (Beare, 2024). CM and hemoglobinuria are signs of retinopathy, requiring regular fundoscopic checks and comprehensive ophthalmoscopic training for healthcare professionals to manage severe cases (Olayinka et al., 2023). Experimental studies on CM in mice reveal that inflammation and bleeding can cause brain injury, affecting critical cognitive regions, and malaria-induced immune responses disrupting balance, leading to neurological impairments like seizures, altered consciousness, psychomotor difficulties, and behavioral abnormalities among other complications such as neurocognitive function, behavior, and thought processes, with coma development occurring gradually in adults (Velagapudi et al., 2019; GBD 2021 Nervous System Disorders Collaborators, 2024).

**Figure 1:**
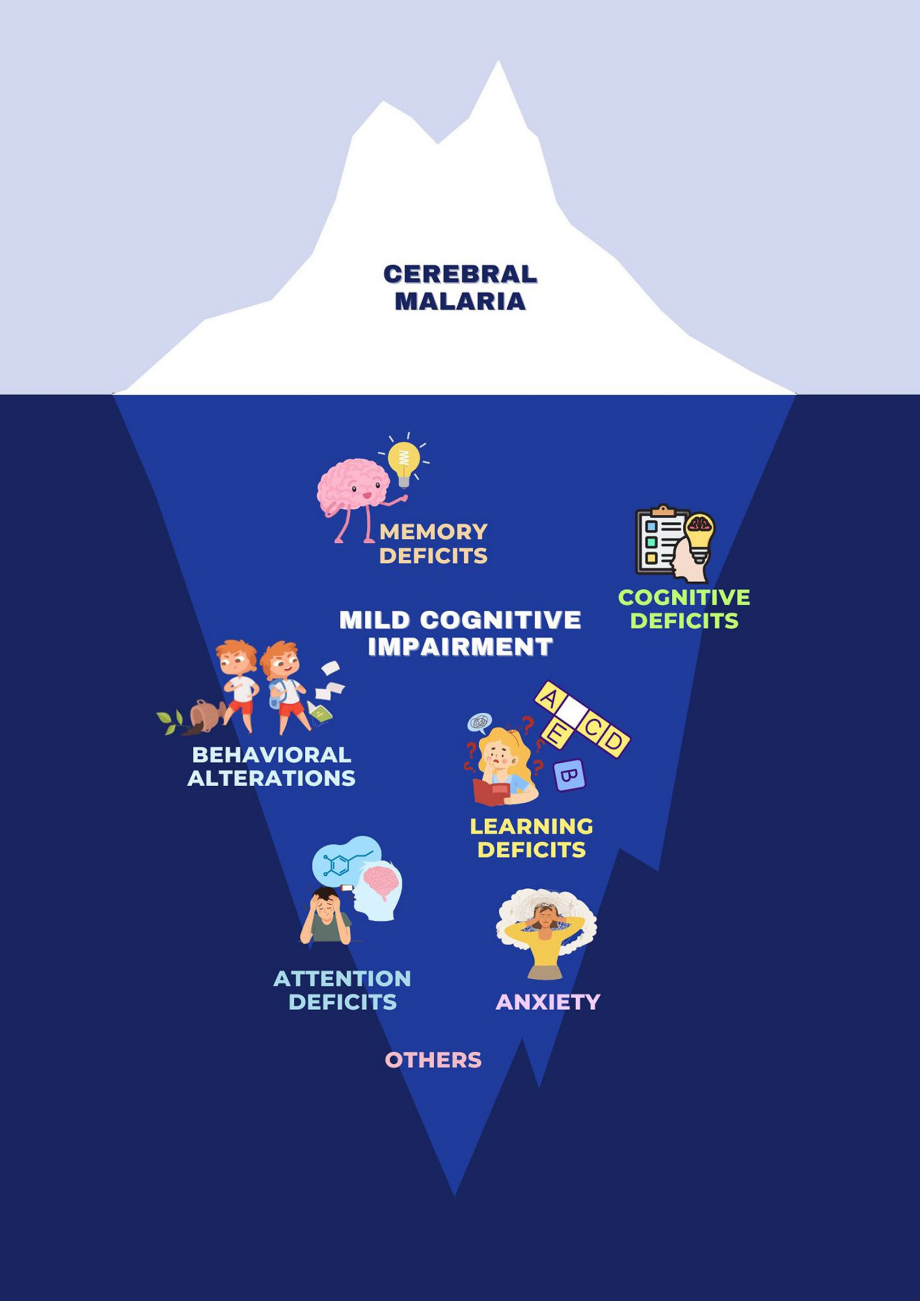
Clinical spectrum of neurological manifestations of malaria. Malaria is commonly associated with a range of physical symptoms and diseases, but it also has significant psychological and neurological impacts. These can include mild cognitive impairment, behavioral alterations, attention deficits, memory deficits, anxiety, learning deficits, and broader cognitive deficits.

## Clinical features of Psychological and Neurological Consequences of malaria

Mental health is more than the absence of mental disorders. It exists on a complex continuum, which is experienced differently from one person to the next, with varying degrees of difficulty and distress and potentially very different social and clinical outcomes (World Health Organization, 2024). Malaria has a complex psychological impact (Tapajós et al., 2019; Pessoa et al., 2022; Nakitende et al., 2023), where both malaria and anti-malarial drugs are linked to neuropsychiatric symptoms, often causing acute anxiety, fear, and obsessive-compulsive behaviors, leading to disruptive actions like aggression and defiance (Okojie et al., 2019; Reddy, 2019; Chaudhry et al., 2022). Guillain-Barré syndrome (GBS), also known as acute inflammatory demyelinating polyneuropathy (AIDP), is a neurological side effect of malaria treatment in patients experiencing aparasitaemic symptoms (Carreira et al., 2019). Post-malaria neurological syndrome (PMNS), a distinct neurological disorder results during severe *P. falciparum* recovery, and is distinct from other post-malaria neurological disorders (Poulet et al., 2019) as it arises within two months after CM, once parasites have cleared with symptoms like confusion, fever, seizures, aphasia, tremors, and myoclonus (Poulet et al., 2019). Opsoclonus-myoclonus syndrome is characterized by repeated jerks and movements of eyes and limbs, occuring as a neurological complication following the acute phase of PMNS (Lev et al., 2020). There may also be a higher chance of dementia associated later in life with cerebral malaria, according to research (Benyumiza et al., 2023). Factors, such as headaches, body aches, sensory symptoms, tiredness, and weakness, also increase susceptibility to mental disorders. About 50% of children admitted to hospitals due to malaria experience neurological complications due to factors like low blood sugar, inflammation, elevated pressure, decreased oxygen levels, and excitotoxicity. Customized evaluations of their performance and patient outcomes can identify key drivers and post-malaria effects (Lima et al., 2021).

This is crucial as some of these symptoms and outcomes are not only limited to CM but are also seen in non severe malaria among both children and adults (Vitor-Silva et al., 2009; Fink et al., 2013; Tapajós et al., 2019; de Sousa et al., 2021).

## Malaria’s impact on cognition

Malaria has a noticeable effect on children’s cognitive development, which is often overlooked as it is still unclear how CM impacts a child’s abilities and functions beyond the aspects related to neurological consequences (Fernando et al., 2010; White, 2022). However, it affects children’s cognitive development, leading to neurological, cognitive, and behavioral issues exacerbated by anemia and malnutrition, especially in low- and middle-income countries (LMICs), due to disruption of brain oxygen delivery and impaired cognition in infected adults which hinders education and employment leading to adverse cognitive and behavioral outcomes, particularly affecting memory, attention, language, and externalizing behavioral issues (Ssemata et al., 2020). Research in Uganda shows that children with severe malarial anemia (SMA) and CM have lower reading scores in comparison to uncomplicated malaria, suggesting post-discharge malaria chemoprevention measures that could improve long-term academic performance (Nakitende et al., 2023). CM and SMA are linked to neuro-developmental impairment in African children, but long-term mental health disorders are not well-understood (Trivedi and Chakravarty, 2022). The impact on cognitive and behavioral abilities leads to poorer performance in one out of four children after more than five episodes compared to those with up to three fever episodes (John et al., 2008; Rosa-Gonçalves et al., 2022).

A study based on children aged 0–18 years showed clinical features like impaired consciousness, respiratory distress, multiple convulsions, prostration, abnormal bleeding, jaundice, severe anemia, hypoglycemia, acidosis, hyperlactatemia, renal impairment, hyperparasitemia, CM, or SMA, often faced neurological impairments, limitations in daily activities, and restrictions in life participation with disability aspects including issues in mental, sensory, and neuromusculoskeletal functions (Engeda et al., 2023). A 2022 study found that Computerized Cognitive Rehabilitation Therapy (CCRT) did not significantly improve academic performance in Ugandan schoolchildren aged five to twelve years, with age, socioeconomic status, gender, and home environment playing significant roles (Miller et al., 2022) while another study evaluating CCRT on children recovering from severe malaria (SM) showed some improvement in learning outcomes, but the intervention group showed lower working memory scores.

In severe cases, being in the hospital also can be stressful for children, making them feel like they have little control over what’s happening to them as illness, injury, and hospital stays can impact a child’s development, including thinking, physical abilities, emotions, and social skills. Such traumatic experiences can lead to immediate or long-term psychological effects which can improve through collaboration with psychologists, human development, and counseling globally (Claridge and Powell, 2023). Establishing childhood disability clinics with interdisciplinary teams could help reduce the prevalence and severity of these disabilities, especially considering malaria’s significant impact (Abdullahi et al., 2022).

## Maternal health risks and consequences of malaria infections in association with mental health

In Sub-Saharan Africa (SSA), malaria is a major cause of illness and death (Datta et al., 2023) particularly during the pregnancy of the first trimester (Accrombessi et al., 2019; Elphinstone et al., 2019; Garrison et al., 2022), with an increased risk of *P. falciparum* infection leading to severe consequences like pregnancy loss (Mahamar et al., 2021), reduced head circumference (Dombrowski et al., 2019), multiple organ dysfunctions (Tripathy et al., 2007), anemia, pulmonary edema, and hypoglycemia due to asymptomatic or low parasitic presence that causes severe consequences with mortality rates reaching up to 50% in the second and third trimesters (Reddy, 2019). Parasitized erythrocytes in the placenta cause maternal immune-inflammatory response, placental pathological changes, poor birth outcomes (Omer et al., 2021). Placental malaria significantly endangers maternal and fetal health and may contribute to impaired neurodevelopment and future developmental disorders (Lawford et al., 2019) which negatively impacts children’s cognitive, behavioral, and executive function (Bangirana et al., 2023) leading to neurodevelopmental delay (Weckman et al., 2021; Garrison et al., 2022) due to increased susceptibility which can significantly affect maternal as well as child health, that leads to higher rates of underestimated factors like anxiety, depression, and PTSD. A case report reveals PM discordance in twins, with a submicroscopic infection in the mother causing worse birth and neurodevelopmental outcomes which raises questions about long-term neurodevelopmental effects from *in utero* infection (Conroy et al., 2019). No studies have yet reported on the neurological functioning of neonates exposed to malaria *in utero* which calls for further studies to evaluate its impact on pregnancy as it is essential to understand the mechanism which will enable healthcare professionals to provide effective postpartum care and develop preventive measures as common psychological challenges like chronic worry, depression, social isolation, financial strain, and self-esteem issues are to be addressed for maintaining well-being by validating tools for accurate diagnosis (Chi et al., 2021; Westby et al., 2021). As mental disorders are highest among illiterate and unemployed individuals, with socioeconomic factors like deprivation and poverty strongly linked to these conditions strengthening these needs at the primary healthcare level is crucial for reducing mental morbidity, as mental health is essential for overall health (Reddy, 2019).

## Implications and importance of mental health services in endemic areas

In India, malaria cases primarily originate from rural and tribal areas (World Health Organization, 2024), with a lack of awareness about the disease despite primary health centers it is a multifactorial problem (Sivanandan et al., 2020; Nema and Ghosh, 2022; Rahi and Sharma, 2022; Kafczyk and Hämel, 2024). This could be a reason why India’s mental disorder prevalence rates are lower than those of Western countries, possibly due to genetic predispositions, family support systems, cultural influences, lifestyles, and coping mechanisms. The scarcity of trained healthcare professionals and traditional beliefs in supernatural powers contribute to delays in diagnosis and treatment which could be bridged by recruiting tribal individuals, training healers and community health workers to understand traditional herbal medicines to strengthen the primary healthcare system (Negi and Singh, 2019; Nema et al., 2019; Kumar et al., 2020) by integrating mental health support into primary healthcare settings as the success of any healthcare intervention program relies on the effective acceptance and utilization of the services provided within the community which in malaria-endemic regions is often limited by factors like household income, proximity to healthcare facilities, and communal beliefs (Singh et al., 2017; Awasthi et al., 2024). However, acceptance of this could significantly assist individuals affected by malaria’s physical and psychological aspects, as it extends beyond individuals, affecting families and communities with increased caregiver burden, reduced productivity, and strained social relationships, particularly in Africa and South Asia among LMICs where research has shown a links malaria to arterial hypertension and depression which increases susceptibility to malaria by affecting immunity and behavior (Jenkins et al., 2017; Volpe and Battistoni, 2019) risking both malaria and mental health disorders impeding treatment and recovery (Jenkins et al., 2019).

## Future research directions

This review, despite the significant lack of research and characterization of malaria and mental health status (Cianconi et al., 2019), highlights the pressing need for further research on the psychological impact of malaria as a successful treatment, particularly for non-severe malaria which requires understanding cognitive and behavioral consequences, enabling targeted therapies (Rosa-Gonçalves et al., 2022). A study reveals that type 2 stimuli-induced immune responses positively impact long-term recognition memory in healthy mice, which confirms the late neurocognitive behavioral dysfunction following non-severe malaria indicating a potential for vaccinations to improve memory weakened by aging and chronic diseases (de Sousa et al., 2021). Among children hospitalized with CM or severe malarial anemia (SMA) with neurologic injury and higher plasma tau levels suggest that plasma tau could be a reliable biomarker for identifying children at risk of persistent neurocognitive impairment (NCI), potentially aiding in acute cognitive rehabilitation (Datta et al., 2021). Despite having the world’s second-largest tribal population, India’s indigenous and tribal communities and their mental health are underdeveloped, and Western psychological tools are not universally applicable, necessitating the development of tailored tools to investigate malaria’s link to psychological distress and evaluate intervention’s effectiveness (Okumu et al., 2022). With the help of the community’s teachers, activists, and volunteers, states must assess this at a tribal and cross-border level by establishing goals using evidence-based public health strategies (Gordon et al., 2019; Lawford et al., 2019; Bangirana et al., 2023) to spread essential lessons about preventing malaria as *P. falciparum* infections can trigger relapses months or even years following the initial infection (de Sousa Pinto et al., 2021; Sato, 2021).

## Discussion

Malaria has a profound impact on endemic regions. Throughout the world, it is characterized by high incidence, mortality, and morbidity rates, with enduring consequences that require integrated management using preventive treatment (Rehman et al., 2019; Cohee et al., 2020), understanding pathogenesis (Bauserman et al., 2019), addressing socioeconomic barriers (Anjorin et al., 2023; Guin et al., 2023; Kooko et al., 2023) and extending vector control measures (Nema et al., 2019; Kafczyk and Hämel, 2024) to support mental health morbidity caused by malaria. Limited healthcare access has to be addressed with the help of advanced and upcoming methods like placental impression smears (Ouédraogo et al., 2019) for early detection of malaria. The accurate diagnosis of malaria is vital given the similarity in symptoms between malaria and other diseases like COVID-19 as there’s a risk of clinicians misdiagnosing one as the other, potentially overlooking the possibility of a co-infection (Gutman et al., 2020; Hussein et al., 2020). In terms of lowering mortality or treatment-related effects such as neurocognitive impairments, current malarial biology therapeutics are still insufficient and (Siddiqui et al., 2020) there is no effective therapy available for addressing the cognitive and behavioral effects resulting from malaria, which necessitates the need for conducting psychological research, developing comprehensive healthcare strategies, and targeted therapies, and collaborating across sectors to address malaria’s impact on cognition. Implementing preventive measures like insecticide-treated bed nets and rapid access to care and effective treatment of uncomplicated malaria is crucial for preventing SM and its complications (Conroy et al., 2019; Boëte et al., 2021).

## Author contributions

PN: Conceptualization, Methodology, Writing – original draft, Writing – review & editing. SBM: Formal analysis, Investigation, Supervision, Validation, Writing – review & editing. SN: Writing – review & editing. GS: Writing – review & editing. PKT: Writing – review & editing. HK: Writing – review & editing. AJ: Writing review & editing. AA: Writing – review & editing. VHC: Conceptualization, Data curation, Formal analysis, Investigation, Methodology, Project administration, Software, Supervision, Validation, Visualization, Writing – original draft, Writing – review & editing.
